# An Open-Label, Randomized, Controlled, Crossover Study to Assess Nicotine Pharmacokinetics and Subjective Effects of the JUUL System with Three Nicotine Concentrations Relative to Combustible Cigarettes in Adult Smokers

**DOI:** 10.1093/ntr/ntab001

**Published:** 2021-01-25

**Authors:** Nicholas I Goldenson, Ian M Fearon, August R Buchhalter, Jack E Henningfield

**Affiliations:** 1 Juul Labs, Inc., Washington, DC, USA; 2 whatIF? Consulting Ltd., Harwell, U.K; 3 PinneyAssociates, Inc., Bethesda, MD, USA

## Abstract

**Introduction:**

This randomized, open-label, crossover clinical study evaluated nicotine pharmacokinetics (PK) and subjective effects of the JUUL System (JS; Juul Labs, Inc.) with three nicotine concentrations compared to the usual brand (UB) cigarettes in 24 adult smokers.

**Methods:**

At five study visits, subjects used either the JS in 59 mg/mL, JS 18 mg/mL (two visits), and JS 9 mg/mL (all tobacco-flavored) or smoked their UB cigarette first during a controlled puffing sequence (CPS) and then ad libitum (5 min) use sessions. Blood samples were taken at specified timepoints for 60 min in each session. The modified Product Evaluation Scale assessed subjective effects 30-min post-use in the CPS session.

**Results:**

Maximum plasma nicotine concentration (*C*_max-BL_), total nicotine exposure (AUC_0-60-BL_), and rate of plasma nicotine rise were significantly lower for all JS products compared to subjects' UB cigarette in CPS and ad libitum use sessions. In both use sessions these PK parameters were significantly higher for JS 59 mg/mL compared to 18 and 9 mg/mL. Subjective measures of cigarette craving relief and “Enough Nicotine” for JS 59 mg/mL did not differ significantly from UB cigarettes, but JS 18 and 9 mg/mL were rated significantly lower than JS 59 mg/mL and UB cigarettes.

**Conclusions:**

Nicotine exposure and subjective relief were directly related to JS nicotine concentration: higher nicotine concentrations gave rise to significantly greater plasma nicotine levels and relief from craving. Heavier and more dependent smokers may require the greater nicotine delivery of JS 59 mg/mL to successfully transition away from cigarettes.

**Implications:**

It has been suggested that electronic nicotine delivery systems (ENDS) and other alternative nicotine delivery products that more closely mimic the nicotine pharmacokinetics (PK) of cigarettes may facilitate smokers transitioning away from cigarettes. We examined nicotine PK and subjective effects of JUUL System (JS) ENDS with three nicotine concentrations (59, 18 and 9 mg/mL) compared to combustible cigarettes. Nicotine delivery from JS ENDS was nicotine concentration dependent, with higher nicotine concentrations giving rise to higher nicotine exposure. These findings suggest that heavier and more dependent smokers may require ENDS with nicotine concentrations greater than 20 mg/mL to successfully transition away from cigarettes.

## Introduction

Cigarette smoking remains the leading cause of preventable disease and death worldwide.^1,2^ While quitting is the most effective means of reducing the harms associated with cigarette smoking,^1^ for smokers who are unwilling or unable to quit, switching to non-combustible nicotine delivery products with lower toxicant exposure may reduce the disease burden of smoking.^2–4^

Aerosol from electronic nicotine delivery systems (ENDS or electronic cigarettes [e-cigarettes]) is believed to contain fewer harmful toxicants and carcinogens than cigarette smoke.^5–7^ Cross-sectional and longitudinal evidence demonstrate that exposure to toxicants is significantly reduced in smokers who switch completely to ENDS,^8–10^ leading some public health bodies, including Public Health England, to promote the use of e-cigarettes as potentially reduced harm alternatives to cigarette smoking for adult smokers.^11^

It has been posited that ENDS and other alternative nicotine delivery products that more closely mimic the nicotine pharmacokinetics (PK) of cigarettes may facilitate smokers transitioning away from smoking.^12,13^ An important motivator of alternative nicotine product use is the ability to reduce cravings for cigarettes and relieve withdrawal symptoms,^14,15^ as increases in craving and withdrawal precede relapse to smoking.^16,17^ Recent evidence also suggests that positive subjective responses to ENDS use, such as satisfaction and reward, may also be associated with increased uptake of ENDS and switching away from cigarette smoking.^18–20^

Previous studies have evaluated nicotine PK and subjective effects of the JUUL System (JS; Juul Labs, Inc.), a closed-system ENDS (ie, uses pods prefilled by the manufacturer with no modifiable settings) with a nicotine-salt (vs. freebase) formulation, with 59 mg/mL nicotine concentration.^13,21,22^ However, per the European Union's Revision of the Tobacco Products Directive (EU TPD) the maximum nicotine concentration for ENDS is 20 mg/mL,^23^ and there are no existing data evaluating JS with nicotine concentrations below this limit. The primary aim of this study was to assess nicotine PK and subjective effects of JS with three nicotine concentrations: 59, 18, and 9 mg/mL, compared to combustible cigarettes among adult smokers. A secondary aim was to evaluate if the use of different wicking materials (silica vs. cotton) to draw the e-liquid from the reservoir to the heating coil of the JS affected nicotine PK and subjective effects.

## Methods

### Study Design

This randomized, open-label, crossover study (ISRCTN 18302793) was conducted from July to August 2019 at the clinical facilities of Celerion, Inc, Belfast, United Kingdom (UK) in accordance with the principles of the International Conference on Harmonisation Harmonised Tripartite Guideline for Good Clinical Practice and the Declaration of Helsinki. The favorable opinion was received from the Health and Social Care Research Ethics Committee B of the Office for Research Ethics Committees (REC), Northern Ireland. All subjects received remuneration for their participation in the study.

On each assessment day, subjects used a randomly assigned study product in a crossover fashion according to a randomization schedule (Latin Square design with a block randomization scheme).

### Subjects

Subjects were adults aged 21–65 years inclusive who were current smokers of at least 10 manufactured, non-mentholated cigarettes a day and had been smoking for at least 12 months. At the screening, which took place 28 days or less before the first assessment day, potential subjects provided written consent and underwent assessments to ensure that they were in good health (eg, review of medical history, physical examination, electrocardiogram [ECG], vital signs measurements). Assessments also included urine cotinine analysis (≥200 ng/mL) and exhaled breath carbon monoxide (eCO) assessment (>10 ppm) to confirm cigarette smoking and a urine screen for drugs of abuse.

Female subjects were ineligible if they were pregnant or breastfeeding, and were required to use contraception for the duration of the study. Exclusion criteria also included any clinically relevant medical or psychiatric disorder, abnormal findings on the physical examination, ECG or clinical laboratory assessments, or a positive screen for alcohol or drugs of abuse. Subjects were not excluded for using ENDS concurrently with cigarettes.

### Study Products

The four JS products used in the study were as follows: (1) 59 mg/mL nicotine Virginia Tobacco flavor (silica wick); (2) 18 mg/mL Golden Tobacco (silica wick); (3) 18 mg/mL Golden Tobacco (cotton wick); and (4) 9 mg/mL Golden Tobacco (cotton wick); pH of the e-liquids ranged from 5.9 to 6.2. All subjects provided their usual brand (UB) cigarette for use as the study reference cigarette.

### Study Procedures

At screening, subjects underwent a brief trial session that involved watching an instructional video and then performing the controlled puffing sequence (CPS) with JS 59 mg/mL to ensure that they were willing and able to use the study products and could perform the CPS. The CPS involved taking a 3-second puff, removing the device from their mouth, and then inhaling for an additional 3 s prior to exhaling. This was repeated every 30 s for a total of 10 inhalations. Potential subjects who were unable to reduce the weight of the pod by 20–60 mg during the CPS were excluded from the study after three attempts.

Subjects who passed all screening assessments and completed the ENDS trial session visited the clinic site on five separate days, during which they used their randomly-assigned study product and completed PK and subjective effects assessments. Prior to each assessment day, subjects were instructed to abstain from the use of any nicotine-containing products for a period of at least 12 hours; compliance was assessed by measuring eCO (<15 ppm). On each study day, subjects first used their randomly-assigned test product according to the CPS. Two hours after collection of the last blood sample, subjects underwent a 5-minute ad libitum use session with the same study product. Individual assessment days were separated by at least 24 hours.

### Nicotine Pharmacokinetics Assessments

For the CPS and ad libitum sessions, 4 mL venous blood samples for nicotine analysis were collected 5-min prior to the first inhalation (-5) and 1.5-, 3-, 5-, 6-, 7-, 8-, 10-, 15-, 30-, and 60-min after the first inhalation. For the sessions that involved cigarette smoking, additional cigarettes were provided if a single cigarette was completed before the end of each session (10 puffs [CPS] or 5 min [ad libitum]). Blood samples were taken using an indwelling catheter and collected in K_2_EDTA Vacutainer tubes. Plasma nicotine analysis was performed as described previously (Study 1) with a lower limit of quantification of 0.2 ng/mL.^24^ Each pod was weighed before and after the CPS and ad libitum use sessions to calculate the change in pod weight and estimate the amount of nicotine consumed.

### Subjective Effects Assessments

A 20-item modified Product Evaluation Scale (mPES)^25^ was completed after the collection of the 30-minute blood sample in the CPS session. All items were answered on seven-point response scales ranging from “not at all” to “extremely.”  ^25^ The mPES consists of four composite subscales: (1) “Satisfaction”; (2) “Psychological Reward”; (3) “Aversion”; and (4) “Relief.” In addition, one individual item of the “Relief” subscale, “Was it enough nicotine?” (“Enough Nicotine”) was analyzed separately.

### Safety Assessments

Safety and tolerability were assessed via incidence and nature of adverse events (AEs) by the study investigator.

### Statistical Analyses

Descriptive statistics for PK parameters including baseline-adjusted maximum plasma nicotine concentration (*C*_max-BL_), time to maximum plasma nicotine concentration (*T*_max_), baseline-adjusted total plasma nicotine uptake at 60-min (AUC_0-60-BL_; calculated with linear trapezoidal method [linear interpolation]), and rate of plasma nicotine rise (*C*_max-BL_ divided by *T*_max_ [slope from baseline to *C*_max-BL_]) were summarised for each study product and use session.

Statistical modeling of PK parameters was conducted within the CPS and ad libitum sessions. Log-transformed *C*_max-BL_ and AUC_0-60-BL_ were included as dependent variables in separate linear mixed-effects models with fixed effects of the product (five study test products), assessment day (Days 1 to 5) and sequence (Sequences 1 to 5) and a random subject term. Back-transformed (exponentiated) least-squares coefficients (geometric mean ratios between the test products) along with two-sided 90% confidence intervals (CIs) for the estimated coefficients were calculated. Statistically significant differences between test products were concluded if 90% CIs did not overlap with 1.00. *T*_max_ was analyzed separately using a Wilcoxon Signed Rank Test, linear mixed-effects models were used to conduct post hoc pairwise comparisons in the rate of plasma nicotine rise.

The mPES subscale and individual item scores were summarized descriptively for each study product; post hoc pairwise comparisons between test products were tested with linear mixed-effects models.

Statistical analyses were performed using SPSS Version 25 (Armonk, NY) with alpha = 0.05 (two-tailed).

## Results

### Study Population

Of 81 subjects who were screened, 35 (43.2%) met eligibility requirements (Supplementary Table 1). Ten eligible subjects were not randomized: nine subjects were not needed as study enrolment had been reached and one subject was discontinued prior to product use on Day 1 due to a positive alcohol breath test. Thus, 25 subjects (20 males and 5 females) were randomized into one of the five product sequences and 24 subjects completed the study per protocol. One subject was discontinued due to a baseline AE (urinary tract infection).

Subjects (mean age [*SD*] = 41.5 [9.93]) were predominantly male (83%) and all were of the white race and not Hispanic or Latino ethnicity (Supplementary Table 2). On average, subjects usually smoked 20.3 cigarettes per day (*SD* = 5.57 [Range: 14–29]), had been smoking for an average of 19.1 years (*SD* = 7.67 [Range: 7–35]). Approximately 88% of subjects reported that their UB cigarette was of a high International Organization for Standardization tar/nicotine/carbon monoxide yield. No subjects reported ever-using ENDS.

### Nicotine Pharmacokinetics

**Figure 1.**
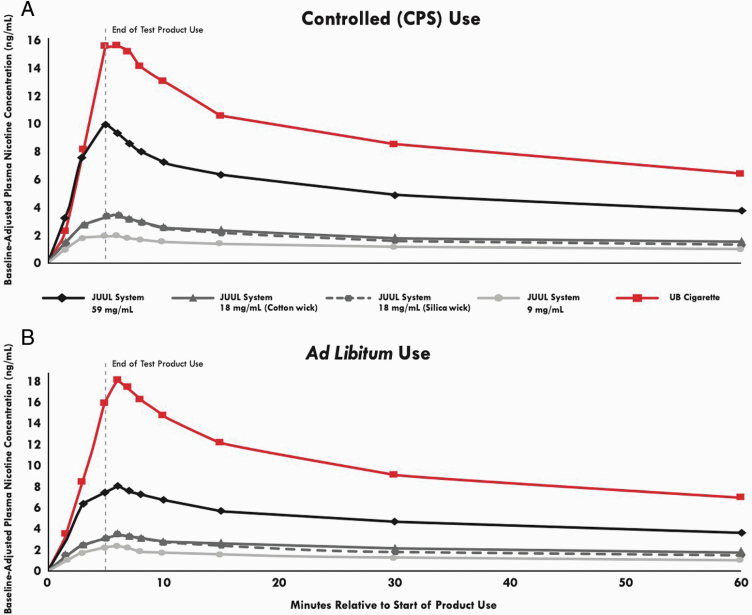
Mean baseline-adjusted nicotine concentration by nominal time in the controlled use (CPS) and ad libitum use sessions. *N* = 23–24 in each case. Errors bars have been omitted for clarity; for variability estimates refer to Table 1.

The time courses of test product plasma nicotine levels over 60 min in both the CPS and ad libitum use sessions are displayed in Figure 1. In both the CPS and ad libitum sessions, aggregated across all datapoints the linear associations (slope) of JS nicotine concentration and *C*_max-BL_ ranged from 0.12 to 0.17 and AUC_0-60-BL_ ranged from 0.07 to 0.08 (*R*^2^ ≥ 0.99; Supplementary Figure 1).

During the CPS, 8 of 24 subjects smoked a second cigarette (Supplementary Table 3). The highest *mean* (±*SD*) *C*_max-BL_ value was observed for UB cigarettes (17.6 ± 8.7 ng/mL; Table 1), which was significantly greater than all JS products (Table 2). Among JS, *C*_max-BL_ was significantly highest for 59 mg/mL (10.6 ± 5.6 ng/mL) followed by 18 mg/mL with silica wick (3.8 ± 2.3 ng/mL), 18 mg/mL with cotton wick (3.7 ± 1.7 ng/mL), and 9 mg/mL (2.4 ± 1.2 ng/mL)—the two JS 18 mg/mL did not differ significantly and were significantly greater than 9 mg/mL (Tables 1 and 2). Similarly, the mean AUC_0-60-BL_ for UB cigarettes was significantly greater than all JS. JS 59 mg/mL was significantly greater than both JS 18 mg/mL and 9 mg/mL; the two JS 18 mg/mL did not differ significantly and were both significantly greater than the 9 mg/mL. The mean rate of plasma nicotine rise was significantly greater for UB cigarettes compared to all JS. JS 59 mg/mL was significantly greater than both 18 and 9 mg/mL products, which did not significantly differ from each other. There were no significant effects of sequence or period (*p* = .12–.84). Mean *T*_max_ was significantly shorter for JS 59 mg/mL (6.2 min) and 18 mg/mL with cotton wick (5.8 min) compared to UB cigarettes (7.8 min), which had the slowest mean *T*_max_ of all test products (Tables 1 and 2).

**Table 1.** PK Parameters of Test Products in Controlled Puffing Sequence (CPS) and Ad Libitum Use Sessions.

PK parameter	JUUL system 59 mg/mL (Silica Wick)	JUUL system 18 mg/mL (Silica Wick)	JUUL system 18 mg/mL (Cotton Wick)	JUUL system 9 mg/mL (Cotton Wick)	UB cigarette
Controlled (CPS) use session					
*C*_max-BL_ (ng/mL)					
Mean (SD)	10.6 (5.6)^a^	3.8 (2.3)^b^	3.7 (1.7)^b^	2.4 (1.2)^c^	17.6 (8.7)^d^
Geometric mean (SD)	9.3 (1.7)	3.2 (1.8)	3.3 (1.6)	2.1 (1.6)	15.7 (1.6)
Median	10.0	3.0	3.5	2.0	14.8
Min to max	2.6 to 29.9	0.5 to 9.6	1.1 to 8.6	0.9 to 5.2	6.5 to 36.5
AUC_0-60-BL_ (h × ng/mL)					
Mean (SD)	5.2 (1.5)^a^	1.8 (0.6)^b^	2.0 (0.7)^b^	1.2 (0.3)^c†^	8.9 (2.7)^d†^
Geometric mean (SD)	5.0 (1.4)	1.7 (1.6)	1.8 (1.4)	1.2 (1.3)	8.5 (1.4)
Median	5.1	1.8	1.9	1.2	9.1
Min to max	1.9 to 8.2	0.3 to 3.4	0.8 to 3.8	0.6 to 1.8	4.6 to 14.8
Rate of plasma nicotine rise (ng/mL per minute)					
Mean (SD)	2.1 (1.5)^a^	0.7 (0.5)^b^	0.8 (0.7)^b^	0.6 (0.5)^b^	2.8 (1.9)^c^
Median	1.7	0.5	0.6	0.3	2.0
Min to max	0.2 to 6.0	0.1 to 1.9	0.2 to 3.0	0.1 to 1.8	0.2 to 7.3
*T*_max_ (min)					
Mean (SD)	6.2 (2.4)^a^	6.3 (1.6)^ab^	5.8 (1.8)^a^	6.6 (5.4)^ab^	7.8 (5.0)^b^
Median	6.0	6.0	6.0	5.6	7.0
Min to max	3.0 to 15.0	2.9 to 10.0	1.6 to 10.0	2.0 to 30.1	5.0 to 30.1
Ad libitum use session					
*C*_max-BL_ (ng/mL)					
Mean (SD)	8.8 (3.2)^a^	3.8 (1.8)^b^	3.7 (1.6)^b^	2.5 (1.0)^c^	20.9 (11.3)^d^
Geometric mean (SD)	8.3 (1.4)	3.5 (1.5)	3.3 (1.7)	2.3 (1.6)	18.4 (1.7)
Median	8.0	3.2	3.5	2.4	17.3
Min to max	4.4 to 15.9	1.0 to 9.8	0.5 to 6.9	0.6 to 4.8	8.3 to 45.3
AUC_0-60-BL_ (h × ng/mL)					
Mean (SD)	4.8 (1.5)^a^	1.9 (0.7)^b†^	2.2 (0.7)^b†^	1.3 (0.4)^c^	9.7 (3.5)^d^
Geometric mean (SD)	4.6 (1.4)	1.8 (1.5)	2.1 (1.4)	1.2 (1.4)	9.2 (1.4)
Median	4.6	1.9	2.1	1.2	8.5
Min to max	1.9 to 7.9	0.5 to 3.3	1.1 to 3.7	0.4 to 1.9	5.4 to 18.3
Rate of plasma nicotine rise (ng/mL per minute)					
Mean (SD)	1.6 (1.0)^a^	0.9 (1.4)^b^	0.7 (0.6)^b^	0.4 (0.3)^b^	3.3 (2.0)^c^
Median	1.3	0.5	0.5	0.4	2.6
Min to max	0.5 to 4.3	0.1 to 6.6	0.1 to 3.2	0.1 to 1.3	1.0 to 8.3
*T*_max_ (min)					
Mean (SD)	6.4 (2.0)^a^	6.5 (2.2)^a^	7.1 (3.2)^a^	6.7 (2.4)^a^	6.7 (1.7)^a^
Median	6.1	6.1	6.1	6.2	6.0
Min to max	3.0 to 10.0	1.5 to 10.8	1.5 to 15.3	3.0 to 15.0	5.0 to 10.0

*N* = 23–24 in each case (^†^*N* = 23). Test product means in the same row that do not share superscripts significantly differ (*p* < .05).

**Table 2.** Statistical Comparison of PK Parameters in the Controlled Puffing Sequence (CPS) and Ad Libitum Use Sessions.

Comparator product	JUUL system 59 mg/mL (Silica Wick)	JUUL system 18 mg/mL (Silica Wick)	JUUL system 18 mg/mL (Cotton Wick)	JUUL system 9 mg/mL (Cotton Wick)
Controlled use session				
*C*_max-BL_ LS means (90% CI)^a^				
JS 18 mg/mL (Silica)	2.88 (2.35, 3.54)	—	1.03 (0.84, 1.27)	0.66 (0.54, 0.81)
JS 18 mg/mL (Cotton)	2.80 (2.28, 3.43)	0.97 (0.79, 1.19)	—	0.64 (0.52, 0.79)
JS 9 mg/mL	4.35 (3.55, 5.35)	1.51 (1.23, 1.85)	1.56 (1.27, 1.91)	—
UB cigarette	0.59 (0.48, 0.73)	0.21 (0.17, 0.25)	0.21 (0.17, 0.26)	0.14 (0.11, 0.17)
AUC_0-60-BL_ LS means 90% (CI)^a^				
JS 18 mg/mL (Silica)	2.98 (2.59, 3.43)	—	1.10 (0.95, 1.27)	0.68 (0.59, 0.79)
JS 18 mg/mL (Cotton)	2.71 (2.35, 3.12)	0.91 (0.79, 1.05)	—	0.62 (0.54, 0.72)
JS 9 mg/mL	4.36 (3.78, 5.03)	1.46 (1.27, 1.69)	1.61 (1.39, 1.86)	—
UB cigarette	0.59 (0.51, 0.68)	0.20 (0.17, 0.23)	0.22 (0.19, 0.25)	0.14 (0.12, 0.16)
*T*_max_ (*p* value)^b^				
JS 18 mg/mL (Silica)	0.62	—	0.24	0.42
JS 18 mg/mL (Cotton)	0.15	0.24	—	0.88
JS 9 mg/mL	0.59	0.42	0.88	—
UB cigarette	0.03	0.12	0.01	0.06
Ad libitum use session				
*C*_max-BL_ LS means (90% CI)^a^				
JS 18 mg/mL (Silica)	2.38 (1.96, 2.89)	—	0.96 (0.79, 1.16)	0.66 (0.54, 0.80)
JS 18 mg/mL (Cotton)	2.48 (2.05, 3.01)	1.04 (0.86, 1.27)	—	0.69 (0.57, 0.83)
JS 9 mg/mL	3.61 (2.98, 4.38)	1.52 (1.25, 1.84)	1.45 (1.20, 1.76)	—
UB cigarette	0.45 (0.37, 0.54)	0.19 (0.16, 0.23)	0.18 (0.15, 0.22)	0.12 (0.10, 0.15)
AUC_0-60-BL_ LS means 90% (CI)^a^				
JS 18 mg/mL (Silica)	2.60 (2.27, 2.97)	—	1.14 (1.00, 1.31)	0.67 (0.59, 0.77)
JS 18 mg/mL (Cotton)	2.28 (1.99, 2.60)	0.88 (0.77, 1.00)	—	0.59 (0.52, 0.67)
JS 9 mg/mL	3.86 (3.38, 4.40)	1.48 (1.30, 1.70)	1.69 (1.48, 1.94)	—
UB cigarette	0.51 (0.44, 0.58)	0.19 (0.17, 0.22)	0.22 (0.19, 0.25)	0.13 (0.12, 0.15)
*T*_max_ (*p* value)^b^				
JS 18 mg/mL (Silica)	0.26	—	0.65	0.91
JS 18 mg/mL (Cotton)	0.23	0.65	—	0.57
JS 9 mg/mL	0.45	0.91	0.57	—
UB cigarette	0.35	0.99	0.26	0.88

*N* = 23–24 in each case. ^a^Back-transformed (exponentiated) least-squares coefficients (geometric mean ratios) and corresponding two-sided 90% CIs between study products were derived using a mixed-effects model. ^b^*p* values were estimated using a paired Wilcoxon (Mann–Whitney) test.

During the ad libitum session, 7 of 24 subjects smoked a second cigarette; mean puff counts during the ad libitum session ranged from 14.0 to 18.7 (Supplementary Table 3). As in the CPS session, the highest mean *C*_max-BL_ value was for UB cigarettes (20.9 ± 11.3 ng/mL; Table 1), which was significantly greater than all JS (Table 2). Among JS, *C*_max-BL_ was significantly highest for 59 mg/mL (8.8 ± 3.2 ng/mL) followed by 18 mg/mL with silica wick (3.8 ± 1.8 ng/mL), 18 mg/mL with cotton wick (3.7 ± 1.6 ng/mL) and 9 mg/mL (2.5 ± 1.0 ng/mL)—the two 18 mg/mL JS did not differ significantly and were significantly greater than 9 mg/mL (Tables 1 and 2). Mean AUC_0-60-BL_ for UB cigarettes was significantly greater than all JS. The JS 59 mg/mL was significantly greater than both 18 mg/mL and 9 mg/mL; the 18 mg/mL products did not significantly differ and were both significantly greater than 9 mg/mL. The mean rate of plasma nicotine rise was significantly greater for UB cigarettes compared to all JS; JS 59 mg/mL was significantly greater than both 18 mg/mL and 9 mg/mL, which did not significantly differ from each other. There were no significant effects of sequence or period (*p* = .41–.85). Mean *T*_max_ values were similar for all study products (ranging from 6.4 min to 7.1 min) and did not significantly differ (Tables 1 and 2).

Net weight and estimated nicotine aerosolized are displayed in Supplementary Table 3.

### Subjective Effects

Subjective effects data are presented in Table 3. For the mPES “Relief” subscale, mean scores for JS 59 mg/mL did not significantly differ from UB cigarettes, and both were significantly greater than JS 18 mg/mL and 9 mg/mL. The two 18 mg/mL JS did not differ significantly; the 18 mg/mL (silica wick) was significantly higher compared to the 9 mg/mL, but the 18 mg/mL (cotton wick) did not differ significantly from 9 mg/mL.

**Table 3.** mPES Composite Subscale and Individual Item Scores in the Controlled Puffing Sequence (CPS) Session.

mPES	JUUL system 59 mg/mL (Silica Wick)	JUUL system 18 mg/mL (Silica Wick)	JUUL system 18 mg/mL (Cotton Wick)	JUUL system 9 mg/mL (Cotton Wick)	UB cigarette
Relief composite score					
Mean (SD)	5.2 (1.0)^a^	4.6 (1.0)^b^	4.3 (1.1)^bc^	3.8 (1.3)^c^	5.6 (1.3)^a^
Median	5.4	4.4	4.5	4.3	6.0
Min to Max	1.8 to 7.0	2.0 to 7.0	1.4 to 6.2	1.0 to 5.6	2.8 to 7.0
Enough nicotine individual item					
Mean (SD)	5.9 (1.3)^a^	4.7 (1.5)^b^	4.7 (1.7)^b^	4.0 (1.6)^c^	6.1 (1.4)^a^
Median	6.0	5.0	5.0	4.0	7.0
Min to max	2.0 to 7.0	1.0 to 7.0	1.0 to 7.0	1.0 to 7.0	1.0 to 7.0
Satisfaction composite score					
Mean (SD)	4.5 (1.3)^a^	4.9 (1.1)^a^	4.6 (1.3)^a^	4.8 (1.1)^a^	5.5 (1.4)^b^
Median	4.8	4.9	4.5	5.0	5.9
Min to max	1.0 to 7.0	2.0 to 7.0	1.3 to 6.8	2.3 to 7.0	2.3 to 7.0
Psychological reward composite score					
Mean (SD)	4.4 (1.1)^acd^	4.3 (1.0)^abcd^	3.9 (1.3)^b^	4.0 (1.2)^a^	4.6 (1.2)^cd^
Median	4.6	4.3	4.2	4.0	4.6
Min to max	1.6 to 6.2	1.6 to 5.8	1.2 to 6.0	1.2 to 6.0	2.8 to 7.0
Aversion composite score					
Mean (SD)	2.4 (1.6)^a^	1.6 (0.7)^bc^	1.8 (0.7)^bc^	1.5 (0.6)^b^	2.1 (1.2)^ac^
Median	1.9	1.3	1.9	1.5	1.8
Min to max	1.0 to 6.5	1.0 to 3.0	1.0 to 3.3	1.0 to 3.0	1.0 to 5.3

*N* = 23–24 in each case. Test products in the same row that do not share superscripts significantly differ (*p* <.05). Post hoc pairwise differences were tested using multi-level linear models. “Was it enough nicotine?” (“Enough Nicotine”) is an item of the “Relief” subscale. All items were answered on seven-point response scales from 1 (“not at all”) to 7 (“extremely”).

For the “Enough Nicotine” item, mean scores for JS 59 mg/mL did not differ significantly from UB cigarettes, and both were significantly greater than JS 18 mg/mL and 9 mg/mL. Mean scores for both JS 18 mg/mL were significantly higher compared to the 9 mg/mL product but did not differ significantly from each other.

For the “Satisfaction” subscale, mean scores for all JS were significantly lower compared to UB cigarettes, none of the JS differed significantly.

For the “Psychological Reward” subscale, mean scores for JS 59 mg/mL and 18 mg/mL (silica wick) did not differ significantly from UB cigarettes. The JS 18 mg/mL (cotton wick) and 9 mg/mL were significantly lower compared to UB cigarettes. The JS 59 mg/mL and 9 mg/mL were significantly greater than 18 mg/mL (cotton wick), and the JS 59 mg/mL, 18 mg/mL (silica wick) and 9 mg/mL did not differ significantly.

For the “Aversion” subscale mean scores, only JS 9 mg/mL was significantly lower compared to UB cigarettes. Among JS, 59 mg/mL was rated significantly higher than both 18 mg/mL and 9 mg/mL; 18 mg/mL and 9 mg/mL did not differ significantly.

### Safety and Tolerability

There were no serious AEs reported in this study, and no subjects were discontinued because of study-emergent AEs. All AEs were considered mild or moderate (Supplementary Table 4).

## Discussion

This clinical laboratory study assessed nicotine PK and subjective effects of JS with 59 mg/mL, 18 mg/mL, and 9 mg/mL nicotine concentrations and two different wicking materials (silica vs. cotton) in comparison to subjects' UB cigarettes. Peak and total nicotine exposure from the JS products evaluated in this study increased linearly as a function of nicotine concentration. Three of the four JS assessed contained nicotine concentrations lower than the 20 mg/mL level mandated for ENDS under the EU TPD.^23^ While previous studies have reported electrical and chemical characteristics and machine yields^26^ of JS marketed in different countries,^27,28^ this manuscript is one of the first to report nicotine PK in human subjects using JS with nicotine concentrations lower than 59 mg/mL which are marketed outside the United States.

In both CPS and ad libitum sessions, nicotine exposure from all JS was significantly less than that of UB cigarettes. As found in previous ENDS research,^29^ nicotine delivery from JS was nicotine concentration-dependent: use of JS 59 mg/mL resulted in significantly higher *C*_max-BL_ and AUC_0-60-BL_ values than 18 mg/mL, which in turn were significantly greater than 9 mg/mL. Consistent with having the same nicotine concentration, the two JS 18 mg/mL with different wicking materials (silica vs. cotton) exhibited similar PK profiles and parameters. In the CPS but not the ad libitum use session, significant differences in mean *T*_max_ between JS 59 mg/mL and 18 mg/mL (cotton wick) and UB cigarettes were observed. Mean *T*_max_ did not differ significantly between JS products, however rate of plasma nicotine rise (ie, speed of nicotine absorption) was significantly higher for JS 59 mg/mL than 18 mg/mL and 9 mg/mL; the JS 18 mg/mL and 9 mg/mL did not differ significantly.

Use of JS 59 mg/mL resulted in subjective relief from cravings (ie, score on the mPES “Relief” composite subscale [eg, “Did it immediately relieve your craving for a cigarette?,” “Did it relieve withdrawal symptoms?”]) that did not differ significantly from that of UB cigarettes. Given the lower nicotine delivery of JS (vs. UB cigarette), the reduction in cigarette craving after use of JS may have also been influenced by non-nicotine sensory effects.^30^ In contrast, JS 18 mg/mL and 9 mg/mL were rated significantly lower than JS 59 mg/mL and UB cigarettes. Similarly, scores for the “Enough Nicotine” item did not differ significantly between JS 59 mg/mL and UB cigarettes, and both were rated significantly greater than JS 18 mg/mL and 9 mg/mL. These findings concord with a study that found that subjective “nicotine delivery,” “taste” and “pleasantness” did not significantly differ between JS 59 mg/mL and UB cigarettes.^13^ Additionally, JS 59 mg/mL was rated significantly higher on the “Aversion” subscale than both 18 mg/mL and 9 mg/mL, likely because of the aversive sensory effects of nicotine.^31,32^

It has been hypothesized that in order for ENDS to be effective substitutes for cigarettes they must provide satisfying and reinforcing effects and relieve urges to smoke.^3,15^ Previous data indicate that highly-dependent smokers benefit from therapy with stronger forms of nicotine gum,^33–35^ which deliver greater amounts of nicotine^36^ and more effectively reduce craving for cigarettes,^37^ and clinical studies from the 1980s and 1990s concluded that 4 mg nicotine gum was more effective than 2 mg gum, which offered little benefit over placebo.^38–40^ Similarly, nicotine patch labeling instructions state that heavier smokers should use higher dose nicotine-delivery patches^41^ based on research showing that lower doses were less effective.^33,34^

The EU TPD states, “This concentration [20 mg/mL] allows for a delivery of nicotine that is comparable to the permitted dose of nicotine derived from a standard cigarette during the time needed to smoke such a cigarette.” ^23^ All of the JS assessed, including 59 mg/mL, delivered significantly less nicotine than smokers' UB cigarette. These findings are consistent with the extant literature, including studies that found that ENDS with 20 mg/mL concentrations provide less than one-third of the nicotine delivered by one tobacco cigarette.^42,43^

The combined PK and subjective effects data from this study suggest that the lower nicotine delivery and subjective relief from JS 18 mg/mL and 9 mg/mL may not be sufficient to help all smokers, particularly heavier and more dependent smokers, transition away from cigarettes. The PK profiles of the JS also indicate that the pharmacological abuse liability of 59 mg/mL is lower than UB cigarettes but higher than 18 and 9 mg/mL, and that the abuse liability of the JS 18 mg/mL is somewhat higher than the 9 mg/mL. Furthermore, the use of all JS under the study conditions was well tolerated by the subjects.

Nicotine PK parameters and nicotine exposure (ie, nicotine aerosolized) from JS with the same nicotine concentration (18 mg/mL) and power characteristics^27,28^ but with a different wicking material (silica vs. cotton) did not differ significantly. This finding contrasts with a recent study that found machine yields of particulate matter and nicotine are approximately two-times greater for JS with a cotton wick compared to JS with a silica wick.^26^ In the current study JS use was circumscribed (ie, 10 controlled puffs or 5 min of ad libitum use), and it is unknown if nicotine PK from JS with different wicking materials would differ over longer periods of use.

The ENDS literature suggests that factors such as puffing topography, aerosol particle size, particle maturation/evolution and particle water content affect the regional deposition of ENDS aerosol,^6,44^ and could explain why greater aerosolization of nicotine and particulate matter did not manifest as greater nicotine delivery in users. For example, a previous study examined nicotine PK in three current users of JS 59 mg/mL who were also cigarette smokers, in which subjects were required to take one puff every 30 s for a total of 15 puffs.^45^ The three subjects varied widely in their nicotine *C*_max_: for one subject, *C*_max_ from JS was much lower than from UB cigarettes; for the second subject, *C*_max_ was higher from JS; for the third subject, *C*_max_ was similar from JS and UB cigarettes. Hence, user behavior (eg, puff volume and duration) may have a strong influence on nicotine PK, as *C*_max_ varied significantly between subjects even when undergoing the same (fixed) puffing protocol.

The extant literature also suggests that there is variability in nicotine PK and subjective effects produced by JS 59 mg/mL, likely related to subjects' ENDS and tobacco use history, use protocols and other factors.^5,6^ The results of a study that assessed nicotine PK of JS 59 mg/mL and UB cigarettes in 18 US smokers with no experience of JS use found that *C*_max_ values for 59 mg/mL in both controlled and ad libitum use sessions were approximately two-fold lower than those for UB cigarettes,^22^ a finding in accordance with the difference between 59 mg/mL and UB cigarettes reported in the current study. In contrast to these findings, a PK study of 20 UK dual users (daily ENDS users who were also occasional smokers) that utilized a similar ad libitum use session found that nicotine PK characteristics (*C*_max_ and AUC_0-30_) did not differ significantly between JS 59 mg/mL and UB cigarettes.^13^ The literature suggests that users of ENDS and cigarettes (dual users) may be more dependent than exclusive users of either ENDS or cigarettes,^46–48^ and subjects in the UK study had extensive histories of smoking.^13^

Subjects' ENDS use status and experience (ie, familiarization with the use of ENDS) in these studies may also contribute to differences in PK. Cross-sectional evidence demonstrates that blood nicotine levels are lower in ENDS-naïve smokers compared to accustomed ENDS users,^42^ suggesting that experience using ENDS may be associated with increased nicotine PK.^7^ Additionally, a longitudinal study examining nicotine PK in smokers before and four weeks after their initial use of ENDS found increases in *C*_max_ and AUC over time.^49^ Future research should examine nicotine PK in smokers at first use of JS and following a familiarisation period to determine if nicotine delivery increases over time as users modify their behavior to titrate to a satisfying level of nicotine.

A small-scale study (*N* = 6) of current pod-based ENDS users (one-third current smokers, 83% JS users) examined nicotine PK of pod-based ENDS containing 59 mg/mL nicotine and various flavors.^21^ Nicotine boost (a metric similar to *C*_max-BL_) ranged from 16 to 42 ng/mL with a mean of approximately 29 ng/mL, values much higher than those observed herein. However, that study used an intensive puffing regime (30 puffs, each puff 20 s apart over a period of 10 min) that was designed and may be more appropriate for cig-a-like ENDS products,^50^ and which likely resulted in the higher nicotine boost values observed.^21^ Furthermore, in that study there was no cigarette comparator to facilitate understanding of how the nicotine PK profiles would have compared under similar puffing conditions.

Strengths of the study include the within-subjects design, inclusion of a combustible cigarette comparator and both controlled and ad libitum use sessions, as well as the evaluation of two wicking materials and nicotine concentrations above and below the EU TPD limit of 20 mg/mL. The generalizability of the findings may be limited by the homogenous sample and subjects' lack of ENDS use history—which was unsurprising given the low penetration of ENDS in the market in which the clinic site is located. Additionally, subjects were not confined to the clinic site, which did not permit comprehensive monitoring of nicotine/tobacco product use during the study, and data on puff topography (puff volume and duration) were not collected.

## Conclusions

Nicotine delivery from JS with 59, 18, and 9 mg/mL nicotine concentrations evaluated in this study were nicotine concentration-dependent, with higher nicotine concentrations giving rise to significantly higher and greater overall nicotine exposure. JS 59 mg/mL delivered significantly greater levels of nicotine and significantly reduced craving and withdrawal compared to JS 18 and 9 mg/mL. The lower nicotine delivery and subjective relief from JS 18 and 9 mg/mL (vs. 59 mg/mL) may vitiate their ability to act as a satisfying alternative to cigarette smoking: heavier and more dependent smokers may require ENDS with nicotine concentrations greater than 20 mg/mL to successfully transition away from cigarettes.

## Supplementary Material

A Contributorship Form detailing each author's specific involvement with this content, as well as any supplementary data, are available online at https://academic.oup.com/ntr.

ntab001_suppl_Supplementary_Materials

ntab001_suppl_Supplementary_Authorship_Form

## Funding

This study was funded by Juul Labs, Inc.

## Declaration of Interests

NG is an employee of Juul Labs, Inc. IF was an employee of Juul Labs, Inc at the time of study conduct and is currently an independent consultant contracted to Juul Labs, Inc to provide scientific support. AB and JH are full-time employees of PinneyAssociates, a consulting firm contracted to JUUL Labs, Inc to provide scientific support.

## References

[1] U.S. Department of Health and Human Services. The Health Consequences of Smoking: 50 Years of Progress: a Report of the Surgeon General. Atlanta: Department of Health and Human Services, Centers for Disease Control and Prevention, National Center for Chronic Disease Prevention and Health Promotion, Office on Smoking and Health; 2014.

[2] U.S. Department of Health and Human Services Medicine. Publications and Reports of the Surgeon General. How Tobacco Smoke Causes Disease: The Biology and Behavioral Basis for Smoking-Attributable Disease: A Report of the Surgeon General. Atlanta (GA): Centers for Disease Control and Prevention (US); 2010. 21452462

[3] Gottlieb S , Zeller M. A nicotine-focused framework for public health. N Engl J Med. 2017;377(12):1111–1114. 28813211 10.1056/NEJMp1707409

[4] Institute of Medicine National Academies of Sciences. Clearing the Smoke - Assessing the Science Base for Tobacco Harm Reduction. Washington. D.C.: The National Academies Press; 2001. 25057541

[5] Hajek P , Etter JF, Benowitz N, Eissenberg T, McRobbie H. Electronic cigarettes: review of use, content, safety, effects on smokers and potential for harm and benefit. Addiction. 2014;109(11):1801–1810. 25078252 10.1111/add.12659 PMC4487785

[6] National Academies of Sciences, Engineering and Medicine England. Public Health Consequences of E-Cigarettes. Washington, DC: The National Academies Press; 2018. 29894118

[7] Fearon IM , Eldridge AC, Gale N, McEwan M, Stiles MF, Round EK. Nicotine pharmacokinetics of electronic cigarettes: a review of the literature. Regul Toxicol Pharmacol. 2018;100:25–34. 30201538 10.1016/j.yrtph.2018.09.004

[8] Goniewicz ML , Smith DM, Edwards KC, et al. Comparison of nicotine and toxicant exposure in users of electronic cigarettes and combustible cigarettes. JAMA Netw Open. 2018;1(8):e185937. 30646298 10.1001/jamanetworkopen.2018.5937 PMC6324349

[9] Shahab L , Goniewicz ML, Blount BC, Brown J, West R. E-Cigarettes and toxin exposure. Ann Intern Med. 2017;167(7):525–526. 28973204 10.7326/L17-0315

[10] Walele T , Bush J, Koch A, Savioz R, Martin C, O'Connell G. Evaluation of the safety profile of an electronic vapour product used for two years by smokers in a real-life setting. Regul Toxicol Pharmacol. 2018;92:226–238. 29248487 10.1016/j.yrtph.2017.12.010

[11] Public Health England Administration. Evidence Review of E-cigarettes and Heated Tobacco Products 2018. A Report Commissioned by Public Health England. London: PHE Publications; 2018.

[12] U.S. Food and Drug Administration Union. *Technical Project Lead Review of IQOS*. 2019; https://www.fda.gov/media/124247/download. Accessed June 26, 2020.

[13] Hajek P , Pittaccio K, Pesola F, Myers Smith K, Phillips-Waller A, Przulj D. Nicotine delivery and users' reactions to Juul compared with cigarettes and other e-cigarette products. Addiction. 2020;115(6):1141–1148. 31994254 10.1111/add.14936 PMC7318270

[14] Baker TB , Piper ME, McCarthy DE, Majeskie MR, Fiore MC. Addiction motivation reformulated: an affective processing model of negative reinforcement. Psychol Rev. 2004;111(1):33–51. 14756584 10.1037/0033-295X.111.1.33

[15] Abrams DB , Glasser AM, Pearson JL, Villanti AC, Collins LK, Niaura RS. Harm minimization and tobacco control: reframing societal views of nicotine use to rapidly save lives. Annu Rev Public Health. 2018;39:193–213. 29323611 10.1146/annurev-publhealth-040617-013849 PMC6942997

[16] Killen JD , Fortmann SP. Craving is associated with smoking relapse: findings from three prospective studies. Exp Clin Psychopharmacol. 1997;5(2):137–142. 9234050 10.1037//1064-1297.5.2.137

[17] Robinson JD , Li L, Chen M, et al. Evaluating the temporal relationships between withdrawal symptoms and smoking relapse. Psychol Addict Behav. 2019;33(2):105–116. 30614717 10.1037/adb0000434 PMC6405298

[18] Pearson JL , Zhou Y, Smiley SL, et al. Intensive longitudinal study of the relationship between cigalike e-cigarette use and cigarette smoking among adult cigarette smokers without immediate plans to quit smoking. Nicotine Tob Res. 2021;21(3):527–534. 10.1093/ntr/ntaa086 PMC7885790 32421191

[19] Gades MS , Petersen A, Meier E, et al. The role of subjective responses in electronic cigarette uptake and substitution in adult smokers. Drug Alcohol Depend. 2020;212:107999. 32409110 10.1016/j.drugalcdep.2020.107999 PMC7315796

[20] Tucker MR , Laugesen M, Bullen C, Grace RC. Predicting short-term uptake of electronic cigarettes: effects of nicotine, subjective effects, and simulated demand. Nicotine Tob Res. 2018;20(10):1265–1271. 29272446 10.1093/ntr/ntx269

[21] Yingst JM , Hrabovsky S, Hobkirk A, Trushin N, Richie JP Jr, Foulds J. Nicotine absorption profile among regular users of a pod-based electronic nicotine delivery system. JAMA Netw Open. 2019;2(11):e1915494. 31730180 10.1001/jamanetworkopen.2019.15494 PMC6902801

[22] Maloney S , Eversole A, Crabtree M, Soule E, Eissenberg T, Breland A. Acute effects of JUUL and IQOS in cigarette smokers. Tob Control. 2020. doi:10.1136/tobaccocontrol-2019-055475 PMC7864587 32041833

[23] The European Parliament and the Council of the European Union. The approximation of the laws, regulations and administrative provisions of the Member States concerning the manufacture, presentation and sale of tobacco and related products and repealing Directive 2001/37/EC. In: The European Parliament and the Council of the European Union, ed. Official Journal of the European Union; 2014

[24] Fearon IM , Eldridge A, Gale N, et al. E-cigarette nicotine delivery: data and learnings from pharmacokinetic studies. Am J Health Behav. 2017;41(1):16–32. 27935787 10.5993/ajhb.41.1.2

[25] Hatsukami DK , Zhang Y, O'Connor RJ, Severson HH. Subjective responses to oral tobacco products: scale validation. Nicotine Tob Res. 2013;15(7):1259–1264. 23239843 10.1093/ntr/nts265 PMC3682844

[26] Mallock N , Trieu HL, Macziol M, et al. Trendy e-cigarettes enter Europe: chemical characterization of JUUL pods and its aerosols. Arch Toxicol. 2020;94(6):1985–1994. 32189038 10.1007/s00204-020-02716-3 PMC7303078

[27] Erythropel HC , Anastas PT, Krishnan-Sarin S, O'Malley SS, Jordt SE, Zimmerman JB. Differences in flavourant levels and synthetic coolant use between USA, EU and Canadian Juul products. Tob Control. 2020. doi:10.1136/tobaccocontrol-2019-055500 PMC7606218 32341193

[28] Talih S , Salman R, El-Hage R, et al. A comparison of the electrical characteristics, liquid composition, and toxicant emissions of JUUL USA and JUUL UK e-cigarettes. Sci Rep. 2020;10(1):7322. 32355323 10.1038/s41598-020-64414-5 PMC7192936

[29] Lopez AA , Hiler MM, Soule EK, et al. Effects of electronic cigarette liquid nicotine concentration on plasma nicotine and puff topography in tobacco cigarette smokers: a preliminary report. Nicotine Tob Res. 2016;18(5):720–723. 26377515 10.1093/ntr/ntv182 PMC5896822

[30] Przulj D , McRobbie H, Hajek P. The effect of sensorimotor replacement on smoking cessation and craving. Open Addict J. 2012;5(1):41–50.

[31] Leventhal A , Cho J, Barrington-Trimis J, Pang R, Schiff S, Kirkpatrick M. Sensory attributes of e-cigarette flavours and nicotine as mediators of interproduct differences in appeal among young adults. Tobacco Control. 2020;29: 679–686. 31852818 10.1136/tobaccocontrol-2019-055172 PMC7473634

[32] Pullicin AJ , Kim H, Brinkman MC, Buehler SS, Clark PI, Lim J. Impacts of nicotine and flavoring on the sensory perception of E-cigarette aerosol. Nicotine Tob Res. 2020;22(5):806–813. 30997500 10.1093/ntr/ntz058 PMC7171271

[33] Stead LF , Perera R, Bullen C, Mant D, Lancaster T. Nicotine replacement therapy for smoking cessation. Cochrane Database Syst Rev. 2008;(1):CD000146. 18253970 10.1002/14651858.CD000146.pub3

[34] Lindson N , Chepkin SC, Ye W, Fanshawe TR, Bullen C, Hartmann-Boyce J. Different doses, durations and modes of delivery of nicotine replacement therapy for smoking cessation. Cochrane Database Syst Rev. 2019;4:CD013308. 30997928 10.1002/14651858.CD013308 PMC6470854

[35] Tobacco Use and Dependence Guideline Panel. Treating Tobacco Use and Dependence: 2008 Update. Vol 53. 2008/09/24 ed. Rockville (MD): US Department of Health and Human Services; 2008.

[36] Hansson A , Rasmussen T, Kraiczi H. Single-dose and multiple-dose pharmacokinetics of nicotine 6 mg gum. Nicotine Tob Res. 2017;19(4):477–483. 27613939 10.1093/ntr/ntw211

[37] Hansson A , Rasmussen T, Perfekt R, Hall E, Kraiczi H. Effect of nicotine 6 mg gum on urges to smoke, a randomized clinical trial. BMC Pharmacol Toxicol. 2019;20(1):69. 31753009 10.1186/s40360-019-0368-9 PMC6873734

[38] Henningfield JE . Nicotine medications for smoking cessation. N Engl J Med. 1995;333(18):1196–1203. 7565976 10.1056/NEJM199511023331807

[39] Tønnesen P , Fryd V, Hansen M, et al. Effect of nicotine chewing gum in combination with group counseling on the cessation of smoking. N Engl J Med. 1988;318(1):15–18. 3336380 10.1056/NEJM198801073180104

[40] Sachs DP . Effectiveness of the 4-mg dose of nicotine polacrilex for the initial treatment of high-dependent smokers. Arch Intern Med. 1995;155(18):1973–1980. 7575051

[41] U.S. Department of Health and Human Services. Smoking Cessation: A Report of the Surgeon General. Rockville, MD: Public Health Service Office of the Surgeon General; 2020.

[42] Farsalinos KE , Spyrou A, Stefopoulos C, et al. Nicotine absorption from electronic cigarette use: comparison between experienced consumers (vapers) and naïve users (smokers). Sci Rep. 2015;5:11269. 26082330 10.1038/srep11269 PMC4469966

[43] Farsalinos KE , Spyrou A, Tsimopoulou K, Stefopoulos C, Romagna G, Voudris V. Nicotine absorption from electronic cigarette use: comparison between first and new-generation devices. Sci Rep. 2014;4:4133. 24569565 10.1038/srep04133 PMC3935206

[44] Zhang Y , Sumner W, Chen DR. *In vitro* particle size distributions in electronic and conventional cigarette aerosols suggest comparable deposition patterns. Nicotine Tob Res. 2013;15(2):501–508. 23042984 10.1093/ntr/nts165

[45] St Helen G , Nardone N, Addo N, et al. Differences in nicotine intake and effects from electronic and combustible cigarettes among dual users. Addiction. 2020;115(4):757–767. 31691397 10.1111/add.14884 PMC7339816

[46] Martinez U , Martinez-Loredo V, Simmons VN, et al. How does smoking and nicotine dependence change after onset of vaping? a retrospective analysis of dual users. Nicotine Tob Res. 2020;22(5):864. 31184754 10.1093/ntr/ntz078 PMC7457319

[47] Rostron BL , Schroeder MJ, Ambrose BK. Dependence symptoms and cessation intentions among US adult daily cigarette, cigar, and e-cigarette users, 2012-2013. BMC Public Health. 2016;16(1):814. 27538489 10.1186/s12889-016-3510-2 PMC4989515

[48] Shiffman S , Sembower MA. Dependence on e-cigarettes and cigarettes in a cross-sectional study of US adults. Addiction. 2020;115(10):1924–1931. 32196810 10.1111/add.15060 PMC7540348

[49] Hajek P , Goniewicz ML, Phillips A, Myers Smith K, West O, McRobbie H. Nicotine intake from electronic cigarettes on initial use and after 4 weeks of regular use. Nicotine Tob Res. 2015;17(2):175–179. 25122503 10.1093/ntr/ntu153 PMC4892703

[50] Yingst JM , Foulds J, Veldheer S, et al. Nicotine absorption during electronic cigarette use among regular users. PLoS One. 2019;14(7):e0220300. 31344110 10.1371/journal.pone.0220300 PMC6657878

