# Supplementary material for: An Open-Label, Randomized, Controlled, Crossover Study to Assess Nicotine Pharmacokinetics and Subjective Effects of the JUUL System with Three Nicotine Concentrations Relative to Combustible Cigarettes in Adult Smokers
Source: Nicotine Tob Res. 2021 Jan 25;23(6):947–55. doi: 10.1093/ntr/ntab001 (PMC8628869; doi:10.1093/ntr/ntab001)
Supplement: ntab001_suppl_Supplementary_Materials [file ntab001_suppl_Supplementary_Materials.docx]

**ONLINE SUPPLEMENTAL MATERIAL**

Figure S1. Linear Associations of JUUL System Nicotine Concentration with C_max-BL_ and

AUC_0-60-BL_

Table S1. Summary of Reasons for Subject Ineligibility

Table S2. Sample Demographics and Tobacco Use Characteristics

Table S3. Test Product Exposure in CPS and *Ad Libitum* Use Sessions

Table S4. Baseline and Study Emergent Adverse Events

**Figure S1.** Linear Associations of JUUL System Nicotine Concentration with C_max-BL_ and AUC_0-60-BL_

*Note*. N=24.

Abbreviations: AUC_-BL_, baseline-adjusted area under the curve; C_max-BL_, baseline-adjusted maximum plasma nicotine concentration; CPS, controlled puff sequence.

**Table S1.** Summary of Reasons for Subject Ineligibility

| Reasons for Ineligibility | N (%) |
| --- | --- |
| Did not provide informed consent | 9 (19.1) |
| Unable to perform controlled puff sequence | 9 (19.1) |
| Positive urine/drug screen | 7 (14.9) |
| Negative response to JUUL System products | 7 (14.9) |
| Unwilling or unable to comply with study requirements | 5 (10.6) |
| Exhaled CO < 10 ppm at screening | 4 (8.5) |
| Unhealthy based on medical history and assessments | 1 (2.1) |
| Clinically relevant medical/psychiatric disorder | 1 (2.1) |
| Clinically significant abnormal physical finding | 1 (2.1) |
| History of diabetes/asthma/COPD | 1 (2.1) |
| Clinically significant laboratory result | 1 (2.1) |
| Positive alcohol breath test at check-in^a^ | 1 (2.1) |

*Note*. N=47.

^a^Met eligibility requirements at Screening but not randomized.

**Table S2.** Sample Demographics and Tobacco Use Characteristics

| **Demographic Characteristics** | N (%) or Mean (*SD*) |
| --- | --- |
| Male Sex | 20 (83.0) |
| Non-Hispanic White Race | 24 (100.0) |
| Age, years, Mean (*SD*) | 41.46 (9.93) |
| BMI (kg/m^2^) | 25.55 (3.34) |
| **Tobacco Use Characteristics** |  |
| No. Cigarettes Smoked per day, Mean (*SD*) | 20.25 (5.57) |
| Duration of smoking, years, Mean (*SD*) | 19.08 (7.67) |
| History of ENDS use | 0 (0.00) |
| Usual Cigarette Brand | |
| Lambert & Butler Original Silver | 5 (20.8)^a^ |
| JPS Real Blue | 4 (16.7)^a^ |
| Mayfair Original Blue | 3 (12.5)^a^ |
| Sterling Original Red | 3 (12.5)^a^ |
| JPS Players Real Red | 2 (8.3)^a^ |
| Benson & Hedges Blue | 1 (8.3)^a^ |
| Benson & Hedges Gold | 1 (4.2)^a^ |
| Regal Filter | 1 (4.2)^a^ |
| Sovereign Blue | 1 (4.2)^a^ |
| Benson & Hedges Sky Blue | 1 (4.2) |
| JPS Superkings Real Blue | 1 (4.2) |
| Chesterfield Blue | 1 (4.2) |

*Note*. N=24.

^a^High International Organization for Standardization tar/nicotine/carbon monoxide yield.

**Table S3.** Product Use in CPS and *Ad Libitum* Use Sessions

|  | JUUL System  59 mg/mL  (Silica Wick) | JUUL System  18 mg/mL  (Silica Wick) | JUUL System  18 mg/mL  (Cotton Wick) | JUUL System  9 mg/mL  (Cotton Wick) | UB Cigarette |
| --- | --- | --- | --- | --- | --- |
| Controlled (CPS) Use | | | | | |
| No. of Puffs, Mean (*SD*) | 10.0 (0.0)^a^ | 10.0 (0.0)^a^ | 10.0 (0.0)^a^ | 10.0 (0.0)^a^ | 10.0 (0.0)^a^ |
| Net Weight Aerosolized (mg), Mean (*SD*) | 25.0 (7.5)^a^ | 26.1 (10.1)^a^ | 27.3 (8.1)^a^ | 31.7 (8.1)^b^ | — |
| Nicotine Aerosolized (mg), Mean (*SD*) | 1.25 (0.37)^a^ | 0.44 (0.17)^b^ | 0.46 (0.14)^b^ | 0.25 (0.06)^c^ | — |
| Smoked one cigarette, N (%) | — | — | — | — | 16 (66.7) |
| Smoked two cigarettes, N (%) | — | — | — | — | 8 (33.3) |
| *Ad Libitum* Use | | | | | |
| No. of Puffs, Mean (*SD*) | 16.0 (5.6)^ac^ | 16.7 (4.3)^ab^ | 17.4 (6.7)^ab^ | 18.7 (9.2)^b^ | 14.0 (4.8)^c^ |
| Net Weight Aerosolized (mg), Mean (*SD*) | 26.3 (9.5)^a^ | 32.4 (12.2)^b^ | 34.9 (15.8)^bc^ | 37.7 (12.6)^c^ |  |
| Nicotine Aerosolized (mg), Mean (*SD*) | 1.31 (0.47)^a^ | 0.55 (0.21)^b^ | 0.59 (0.27)^b^ | 0.30 (0.10)^c^ | — |
| Smoked one cigarette, N (%) | — | — | — | — | 17 (70.8) |
| Smoked two cigarettes, N (%) | — | — | — | — | 7 (29.2) |

*Note*. N=24.

Test products that do not share the same superscript significantly differ (*p*<0.05).

Net Weight Aerosolized (mg) = [Pre-Weight (g) - Post-Weight (g)] × 10^3^.

Nicotine Aerosolized (mg) = Nicotine concentration (5.0%, 1.7%, or 0.8%) × Net Weight (mg).

A total of 12 subjects (50%) smoked 1 cigarette in both the CPS and *ad libitum* sessions, five subjects (20.8%) smoked two cigarettes in the CPS and 1 cigarette in the *ad libitum* session, four subjects (16.7%) smoked 1 cigarette in the CPS and 2 cigarettes in the *ad libitum* session and three subjects (12.5%) smoked 2 cigarettes in both the CPS and *ad libitum* sessions.

Table S4. Baseline and Study Emergent Adverse Events

|  | Baseline | JUUL System  59 mg/mL  (Silica Wick) | JUUL System 18 mg/mL  (Silica Wick) | JUUL System 18 mg/mL  (Cotton Wick) | JUUL System 9 mg/mL  (Cotton Wick) | UB Cigarette |
| --- | --- | --- | --- | --- | --- | --- |
| Adverse Events (AEs) |  |  |  |  |  |  |
| Yes | 1 (4.0%) | 2 (8.3%) | 1 (4.2%) | 0 (0%) | 0 (0%) | 4 (16.7%) |
| No | 24 (96.0%) | 22 (91.7%) | 23 (95.8%) | 24 (100%) | 24 (100%) | 20 (83.3%) |
| Total | 25 (100%) | 24 (100%) | 24 (100%) | 24 (100%) | 24 (100%) | 24 (100%) |
| Serious AEs | 0 | 0 | 0 | 0 | 0 | 0 |
| Yes (Death) | 0 | 0 | 0 | 0 | 0 | 0 |
| Yes (Life Threatening) | 0 | 0 | 0 | 0 | 0 | 0 |
| Yes (Hospitalization) | 0 | 0 | 0 | 0 | 0 | 0 |
| Yes (Disabling) | 0 | 0 | 0 | 0 | 0 | 0 |
| Yes (Birth Defect) | 0 | 0 | 0 | 0 | 0 | 0 |
| Yes (Medically Important) | 0 | 0 | 0 | 0 | 0 | 0 |
| No | 25 (100%) | 24 (100%) | 24 (100%) | 24 (100%) | 24 (100%) | 24 (100%) |
| Total | 25 (100%) | 24 (100%) | 24 (100%) | 24 (100%) | 24 (100%) | 24 (100%) |
| Maximum Intensity |  |  |  |  |  |  |
| Severe | 0 | 0 | 0 | 0 | 0 | 0 |
| Moderate | 1 (4.0%) | 0 | 1 (4.2%) | 0 | 0 | 2 (8.3%) |
| Mild | 0 | 2 (8.3%) | 0 | 0 | 0 | 2 (8.3%) |
| No AE | 24 (96.0%) | 22 (91.7%) | 23 (95.8%) | 24 (100%) | 24 (100%) | 20 (83.3%) |
| Total | 25 (100%) | 24 (100%) | 24 (100%) | 24 (100%) | 24 (100%) | 24 (100%) |
| Worst Relationship |  |  |  |  |  |  |
| Related | 0 | 2 (8.3%) | 0 | 0 | 0 | 3 (12.5%) |
| Not Related | 1 (4.0%) | 0 | 1 (4.2%) | 0 | 0 | 1 (4.2%) |
| No AE | 24 (96.0%) | 22 (91.7%) | 23 (95.8%) | 24 (100%) | 24 (100%) | 20 (83.3%) |
| Total | 25 (100%) | 24 (100%) | 24 (100%) | 24 (100%) | 24 (100%) | 24 (100%) |
| Outcome of AE |  |  |  |  |  |  |
| Fatal | 0 | 0 | 0 | 0 | 0 | 0 |
| Not Resolved | 0 | 0 | 0 | 0 | 0 | 0 |
| Resolving | 0 | 0 | 0 | 0 | 0 | 0 |
| Resolved w/ Sequelae | 0 | 0 | 0 | 0 | 0 | 0 |
| Resolved | 1 (4.0%) | 2 (8.3%) | 1 (4.2%) | 0 | 0 | 4 (16.7%) |
| Unknown | 0 | 0 | 0 | 0 | 0 | 0 |
| No AE | 24 (96.0%) | 22 (91.7%) | 23 (95.8%) | 24 (100%) | 24 (100%) | 20 (83.3%) |
| Total | 25 (100%) | 24 (100%) | 24 (100%) | 24 (100%) | 24 (100%) | 24 (100%) |
